# Supplementary material for: The MBW complex regulates floral volatiles in petunia: transcriptional repressor EOBV is a MYB component of the complex
Source: Plant Physiol. 2026 Oct 20;202(3):kiag777. doi: 10.1093/plphys/kiag777 (PMC13729120; doi:10.1093/plphys/kiag777)
Supplement: Supplementary file 1 [file kiag777_supplementary_data.zip › SupplementaryFigures.pdf]

Supplementary Figures

The MBW complex regulates floral volatiles in petunia: transcriptional repressor EOBV is a MYB component of the complex

Oded Skaliter[1], Raz Cohen[1], Ehud Leor-Librach[1], Ekaterina Shor[1,#], Ori Rudich[1], Orit Edelbaum[1], Elena Shklarman[1], Tania Masci[1] and Alexander Vainstein[1,*]

[1]*Institute of Plant Sciences and Genetics in Agriculture, The Robert H. Smith Faculty of Agriculture, Food and Environment, The Hebrew University of Jerusalem, Rehovot 7610001, Israel*

[#]*Present address: Department of Vegetable and Field Crops, Institute of Plant Sciences, Agricultural Research Organization – Volcani Institute, Rishon LeZion 7505101, Israel*

a

AN1:
GGTAGAGAGGTGTGATTGTGGGGAACAACTCCTGTATTAACGGT
CTTTTCTGATATACAGGTGACTTGTCTAACGTGTCGCAAGGCATT
GTACCAAACGTCGACGTAATAATAAACATGACGTTGGTCTTTGAC
GTGCTATTATAACCTTTCCTCTAATGAAATAAGATGTGGATTATTAA
TATCACTGATTCTTTTTATTTGTATAATCAACTAGTTGTTTAC

b

AN11:
TATGCTCTTTTGCCACACAGAATAAGTTGTAACTATCTAGTAGTAGCAGCAGAAGCAAT
AGAACCTATCTTACATGAAAAAGATTCAATCTTTCAATCACCTTATACTTTAAGCAATTG
CAACTTGTTAGAAAATGCAATAGCAATCCAATCACGCTGCGCAGGAGACCACTGAAGC
TGATTAATCTCAGCACCAGCAGAGTACATCGACATGGGATCAATCCCATTAGGCCCAG
CAACAGTAGGCAACTCCCAAATCAACGCCTGCCCATCATCTCCACCTGAACAAATATG
TCTACAACTCTGGGGAGCCCAAGCAATGGCATTCACACT

c

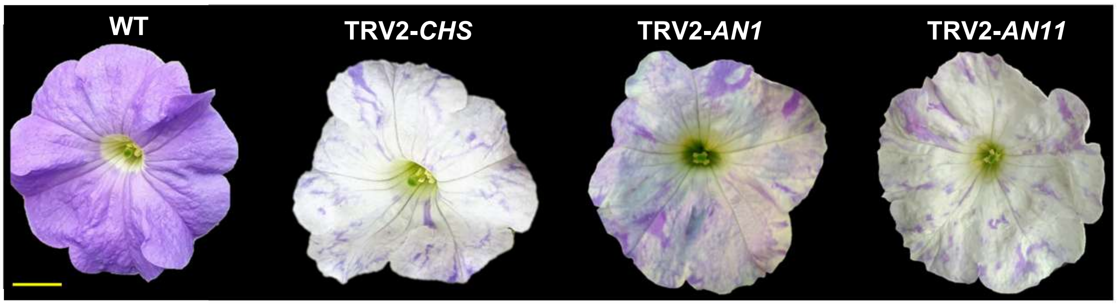

Figure S1. Virus-induced gene silencing (VIGS) of *AN1* and *AN11* in petunia. (a and b) Sequences used for VIGS of 5' untranslated region (UTR) of *AN1* **(a)** and 3' UTR of *AN11* **(b)**. **c)** Representative photos of wild-type (WT) petunia cv. P720 flower, and corollas displaying white sectors due to loss of anthocyanins as a result of *CHS*, *AN1* or *AN11* suppression. Bar = 1 cm.

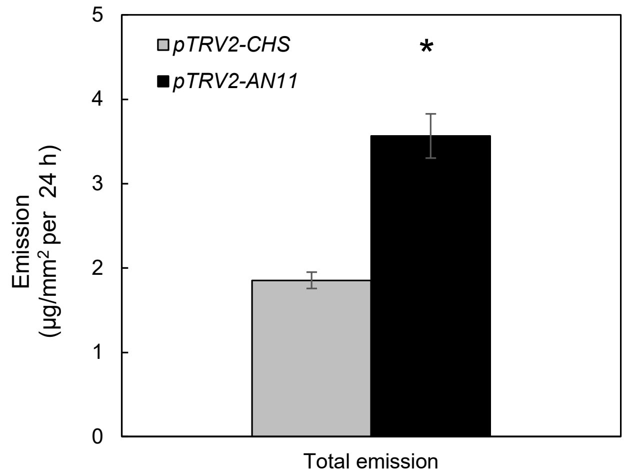

Figure S2. Virus-induced gene silencing (VIGS) of AN11 in petunia P720 results in enhanced emission of volatiles. Petunia flowers were inoculated with *Agrobacterium* carrying pTRV2-CHS as a control or pTRV2-AN11. Flowers were harvested 2 days after inoculation and subjected to localized headspace sampling followed by GC–MS analysis. Data are means ± SEM (n = 4). Significance of differences between treatments was calculated by two-tailed unpaired Student's t-test: *$P \leq 0.05$. Standard errors are indicated by vertical lines.

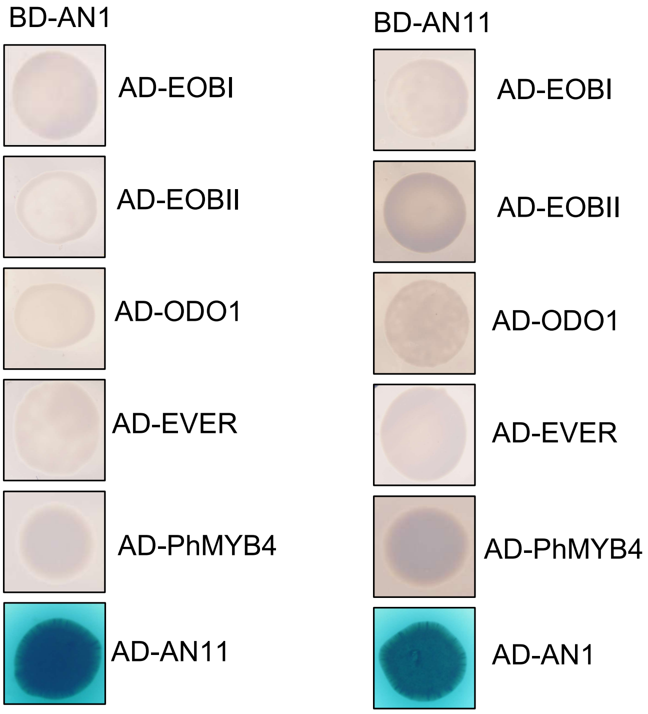

Figure S3. Yeast two-hybrid assay testing the interactions of various MYB regulators of volatile production with AN1 and AN11. The coding sequences of the targets were fused to GAL4 binding domain (BD) or GAL4 activation domain (AD) and transformed into yeast. X-Gal staining was performed on transformed yeast to detect *β*-galactosidase activity, indicating an interaction between bait and prey. The interaction between AN1 and AN11 was used as a positive control. EOBI, EMISSION OF BENZENOIDS I; EOBII, EMISSION OF BENZENOIDS II; ODO1, ODORANT1; EVER, EPIDERMIS VOLATILE EMISSION REGULATOR.

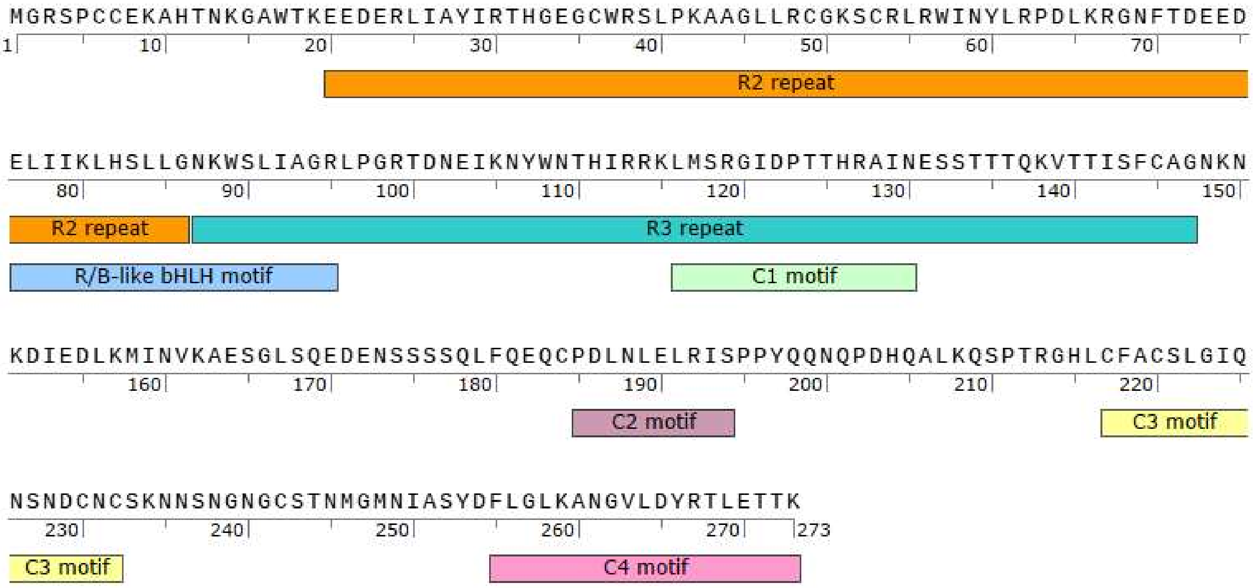

Figure S4. Protein domain analysis of EOBV. The analysis was performed using InterPro (Blum et al., 2021). Domains are color-keyed. R/B-like bHLH motif – [D/E]Lx2[R/K]x3Lx6Lx3R; C1 motif – LlsrGIDPxT/sHRxI/L; C2 motif – pdLNLD/ELxiG/S; C3 motif – CX1-2CX7-12CX2C; C4 motif – FLGLx4-7V/LLD/GF/YR/Sx1LEMK.

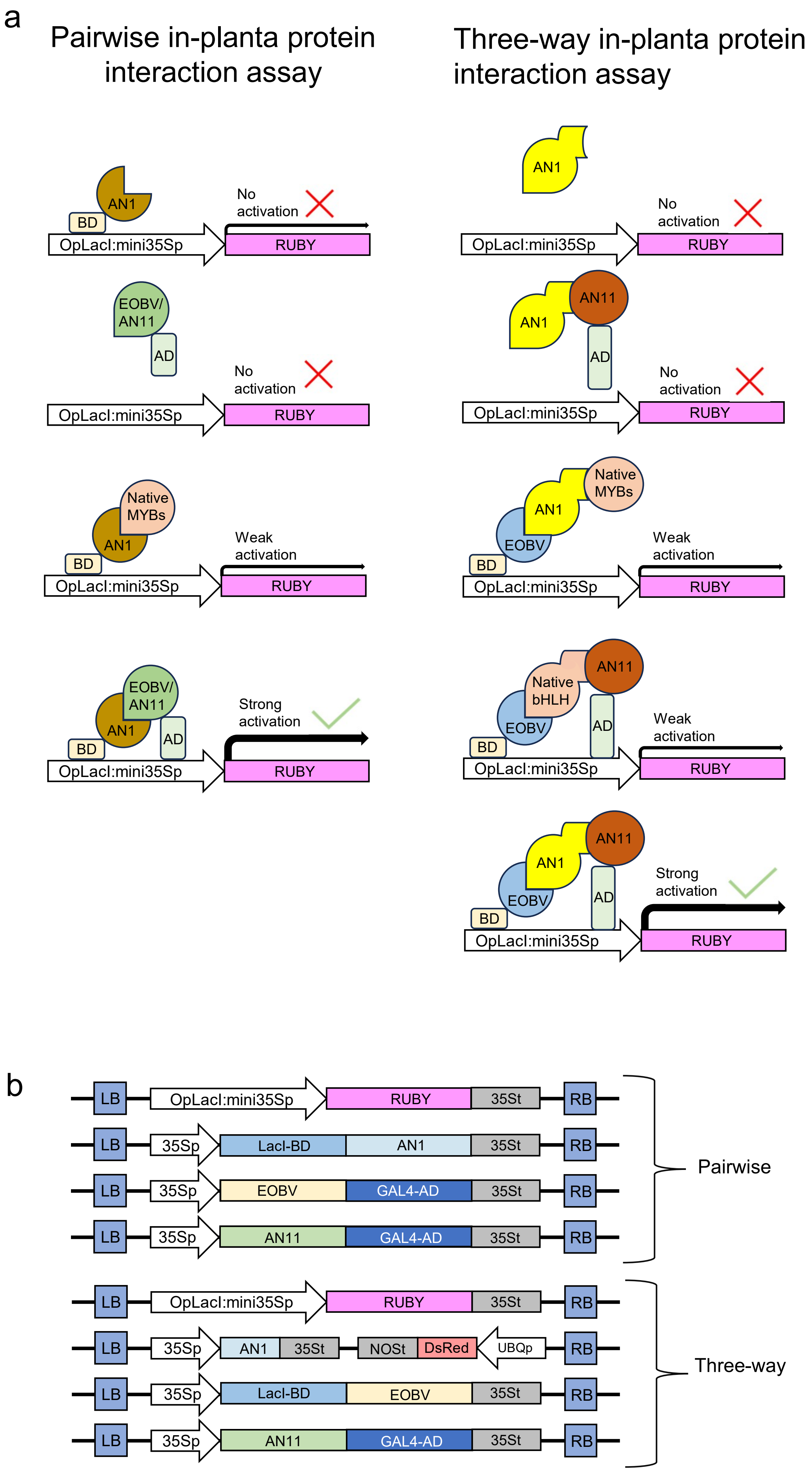
a
Pairwise in-planta protein interaction assay
Three-way in-planta protein interaction assay
AN1
BD
OpLacI:mini35Sp
RUBY
No activation
EOBV/ AN11
AD
Native MYBs
Weak activation
Strong activation
AN11
EOBV
Native bHLH
b
LB
RB
RUBY
35St
35Sp
LacI-BD
EOBV
GAL4-AD
NOSt
DsRed
UBQp
Pairwise
Three-way

c

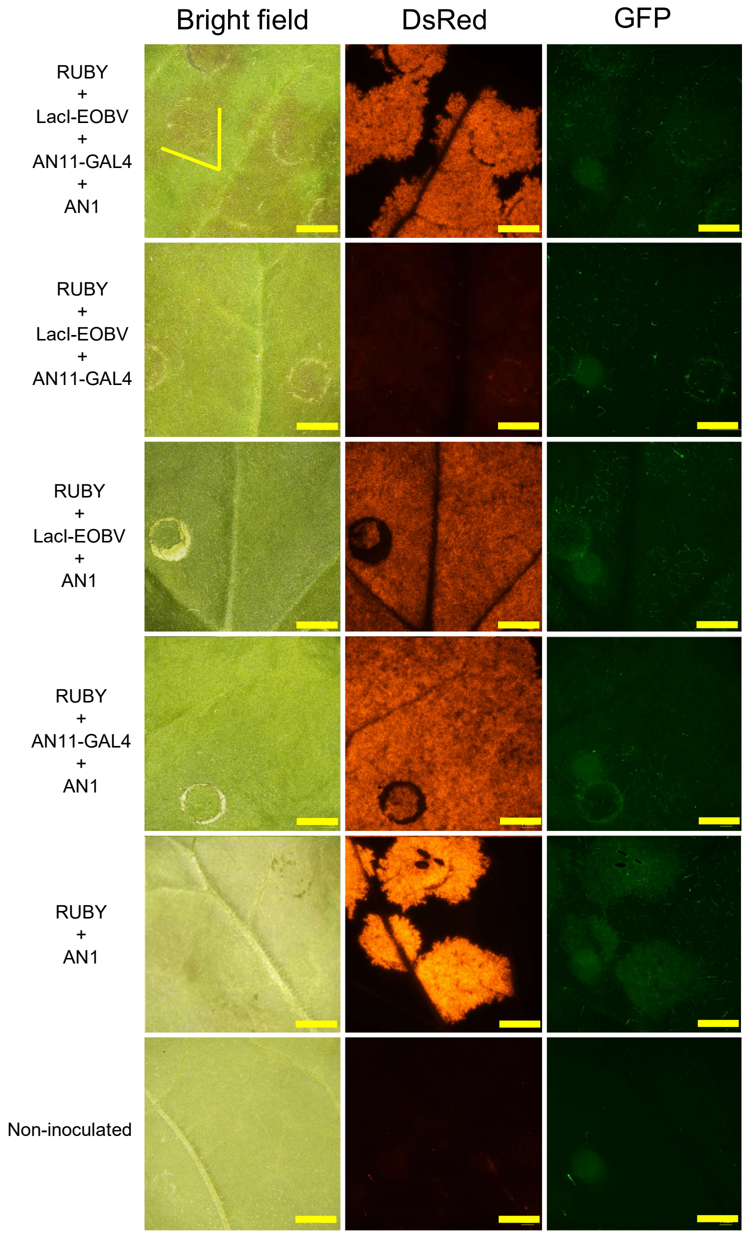

d

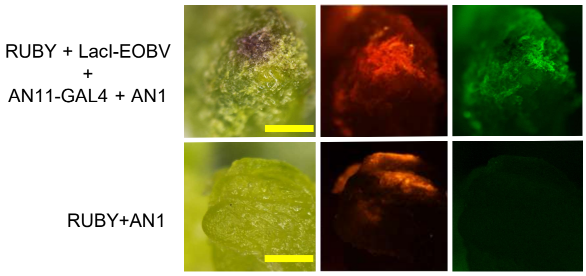

Figure S5. EOBV participates in the MBW complex. a) Schematic model illustrating the in-planta RUBY-based pairwise and three-way protein interaction assays. **b)** Schematic representation of vectors used in the protein–protein interaction assays. Top: vectors used in pairwise interaction assays. Bottom: vectors used in three-way interaction assays. OpLacI:mini35S, 6 repeats of the lac operator fused to a minimal 35S promoter. The coding sequences of the targets were fused to LacI binding domain (BD) or GAL4 activation domain (AD). **c)** *Nicotiana benthamiana* leaves were inoculated with a mixture of agrobacteria carrying different vector combinations; 3 days postinoculation, leaves were examined under a fluorescent stereomicroscope. Yellow arrows mark observable betalain accumulation in leaves inoculated with 6×OpLacI:mini35S:*RUBY*, 35Sp:*LacI-EOBV*, 35Sp:*AN11-GAL4* and 35Sp:*AN1*. Bar = 1 mm. **d)** Representative photos of pigmented regions formed on *Nicotiana tabacum* leaf disks inoculated with 6×OpLacI:mini35S:*RUBY*, 35Sp:*LacI-EOBV*, 35Sp:*AN11-GAL4* and 35Sp:*AN1* and control inoculated with 6×OpLacI:mini35S:*RUBY* and 35Sp:*AN1*. Bar = 1 mm.

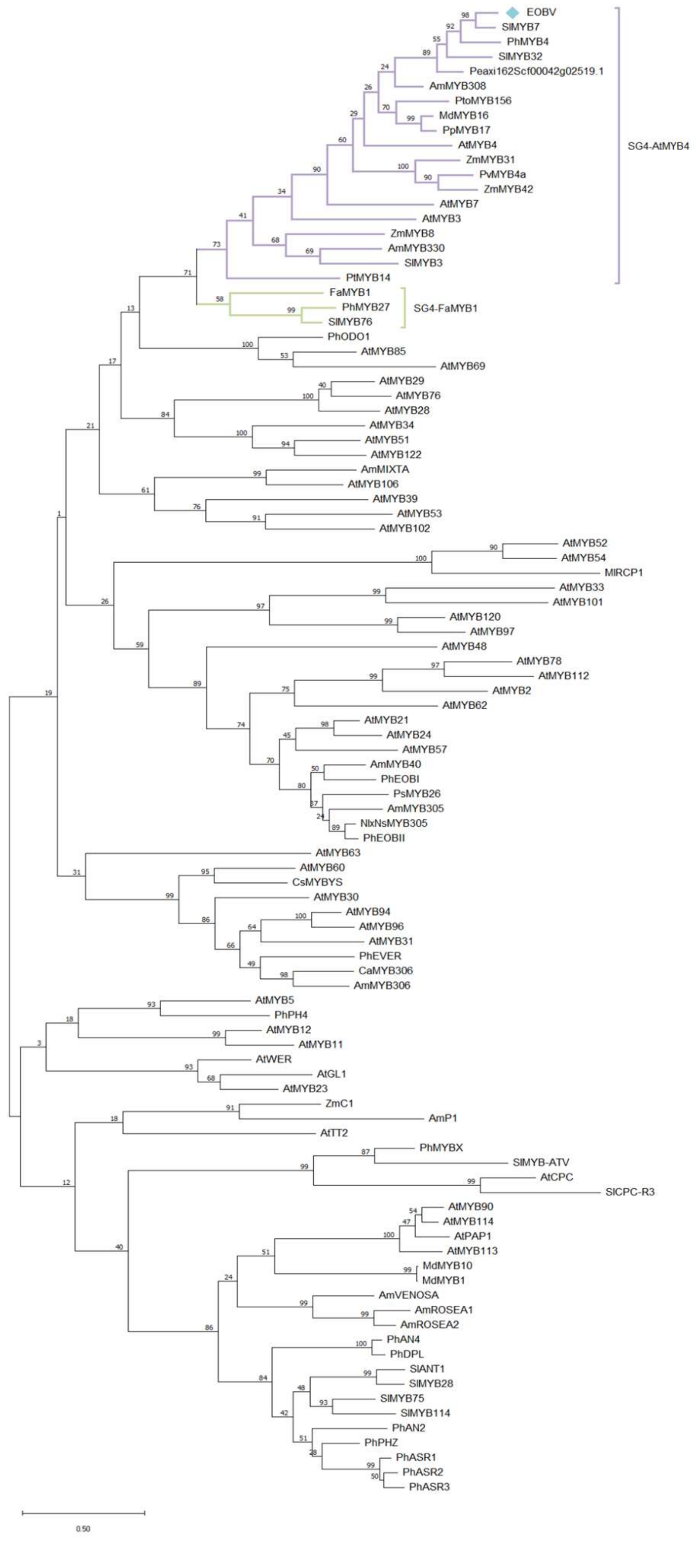

Figure S6. EOBV is an R2R3-MYB subgroup 4 member. Phylogenetic analysis representing the relationships between EOBV (marked by a diamond) and R2R3-MYBs from petunia and other plant species. Subgrouping is based on Dubos et al. (2010). Protein sequence alignment was done by ClustalW followed by maximum-likelihood method with 1000 bootstraps using MEGA version 11. Bootstrap values are indicated at the branch nodes, and scale bar indicates the number of amino acid substitutions per site.

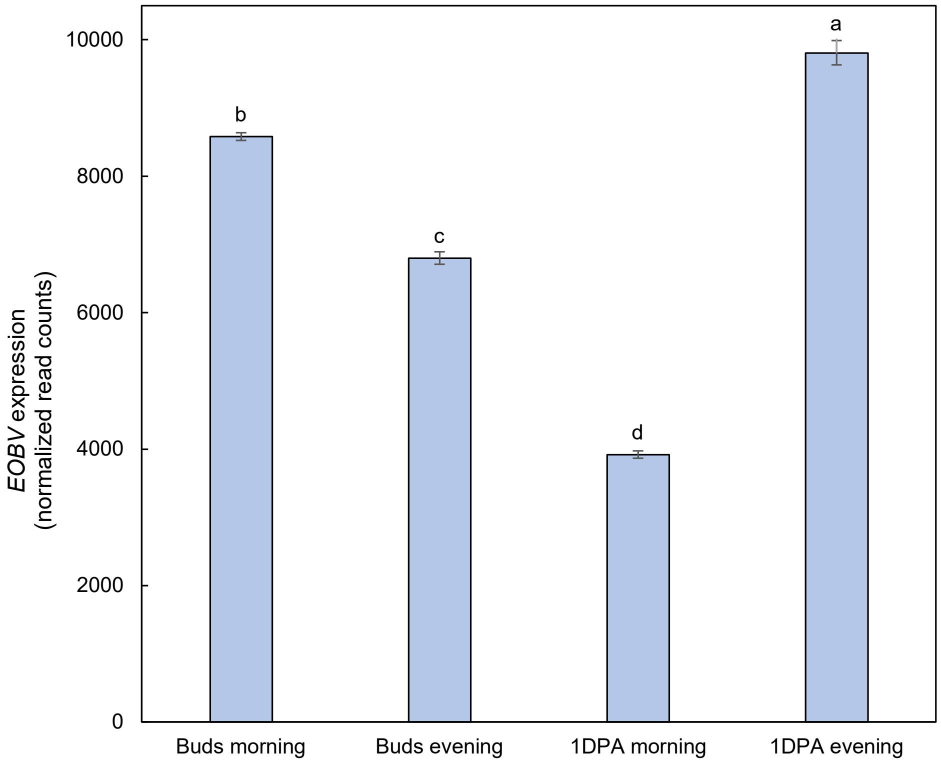

Figure S7. *EOBV* expression levels in petunia floral buds and flowers 1 day postanthesis (1DPA) in the morning (1000 h) and evening (1900 h). Transcripts were detected by RNA-sequencing performed in Shor et al. (2023b). Data are means ± SEM (n = 3). Significance of differences was calculated by Tukey's multiple comparison test following

ANOVA. Values with different letters are significantly different at $P \leq 0.05$. Standard errors are indicated by vertical lines.

a

AAAACAGAAACCAAAGAACAGAGAGGAAGAAAAGAATATACAAGGAATAGGATCAT
GAGTCGAGGTATTGACCCTACAACACACAGGGCTATTAACGAGTCTAGTACTACCACAC
AAAAAGTTACAACAATTTCTTTTTGTGCTGGAAATAAAAATAAAGATATTGAAGATCTG
AAGATGATCAATGTCAAAGCTGAATCTGGACTTAGCCAAGAAGATGAAAATAGTAGCA
GCAGCCAATTATTTCAAGAACAGTGTCCTGATTTGAATCTTGAGCTCAGAATTAGCCCT
CCTTACCAACAAAACCAACCAGATCACCAAGCTTTGAAACAAAGTCCAACAAGGGGC
CATTTGTGTTTTGCATGCAGTTTGGGTATCCAAAACAGTAACGATTGCAATTGCAGTAA
AAATAATAGTAATGGGAATGGTTGCAGTACTAACATGGGTATGAATATTGCAAGTTATG
ATTTTTTAGGATTAAAAGCTAATGGTGTTTTGGACTACAGAACCTTGGAGACTACTAAG
TGATTATTATGTTGGATTACATAAAAAAAAAGAGAATTGAGAAAGACAGTGTTATTAAT
TTAAGTCTTTTTCGAATTTCTCCTATTTGTAAAGTTGAAAGTAATATATAGTAGTATTACA
TTAAGTTGAAAGCAGTACATCAGTGTACCTTATACTAAATTTTAATT

b

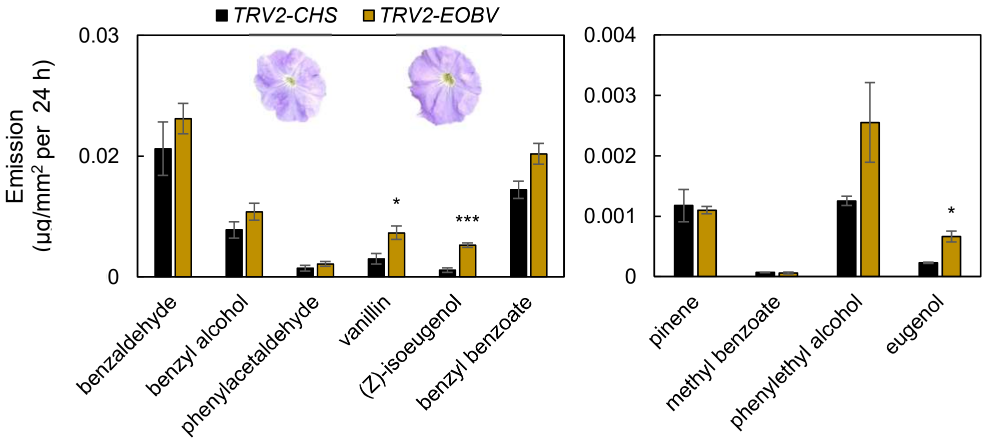

c

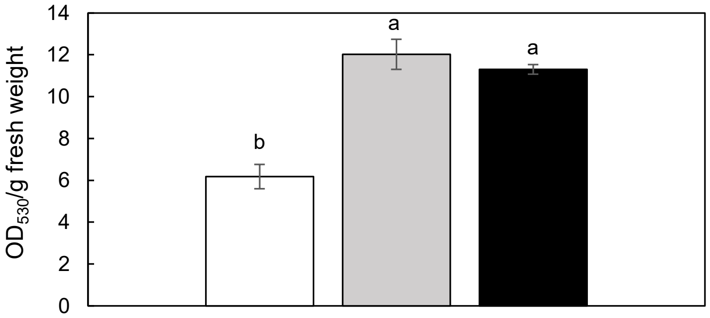

Figure S8. Virus-induced gene silencing (VIGS) of *EOBV* in petunia flowers. a) mRNA sequence of *EOBV*. Red nucleotides, 5' untranslated region (UTR); black nucleotides, coding sequence; green nucleotides, 3' UTR used for VIGS. **b**) Petunia cv. P720 flowers were inoculated with agrobacteria carrying pTRV2-*EOBV* or pTRV2-*CHS* as a control. Flowers were harvested 2 days after inoculation and subjected to localized headspace followed by GC–MS analyses. Data are means ± SEM (n = 5). Significance of differences between treatments was calculated by two-tailed unpaired Student's t-test: *P ≤ 0.05, **P ≤ 0.01, ***P ≤ 0.001. **c)** Anthocyanin content in corollas of petunia cv. Classic Blue Ray accumulating purple anthocyanins following suppression of *EOBV*. Plants (1 month old) were inoculated with agrobacteria carrying pTRV2-*CHS* as a control or pTRV2-*EOBV*. After 1 month, white petal tissue (indicating *CHS* silencing, white bar), non-suppressed purple petal tissue (gray bar) and petal tissue of plants inoculated with TRV2-*EOBV* (black bar) were collected, and anthocyanins were extracted and analyzed spectrophotometrically. Data are means ± SEM (n = 4). Significance of differences was calculated by Tukey's multiple comparison test following one-way ANOVA. Values with different letters are significantly different at P ≤ 0.05. Standard errors are indicated by vertical lines.

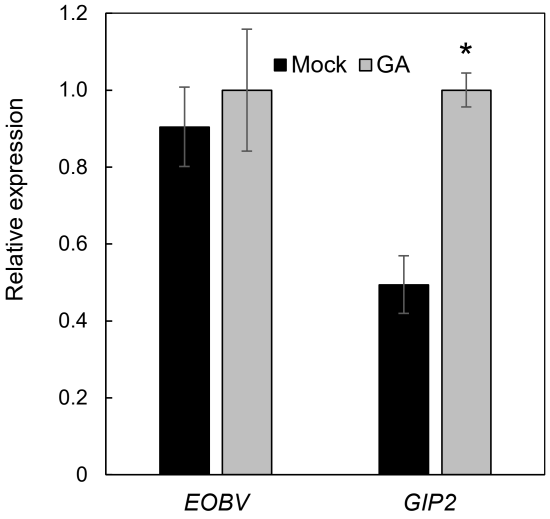

Figure S9. Effect of exogenous gibberellin (GA) application on *EOBV* transcripts. Petunia buds were treated with or without (mock) GA_3 and after 48 h, RNA was extracted from the flowers and subjected to RT-qPCR analyses. Data were normalized to *UBIQUITIN* and to the sample with the highest expression level. Data are means ± SEM (n = 3). Significance of differences between treatments was calculated by two-tailed unpaired Student's t-test: $*P \leq 0.05$. Standard errors are indicated by vertical lines. GIP2, GIBBERELLIN-INDUCED PROTEIN 2.

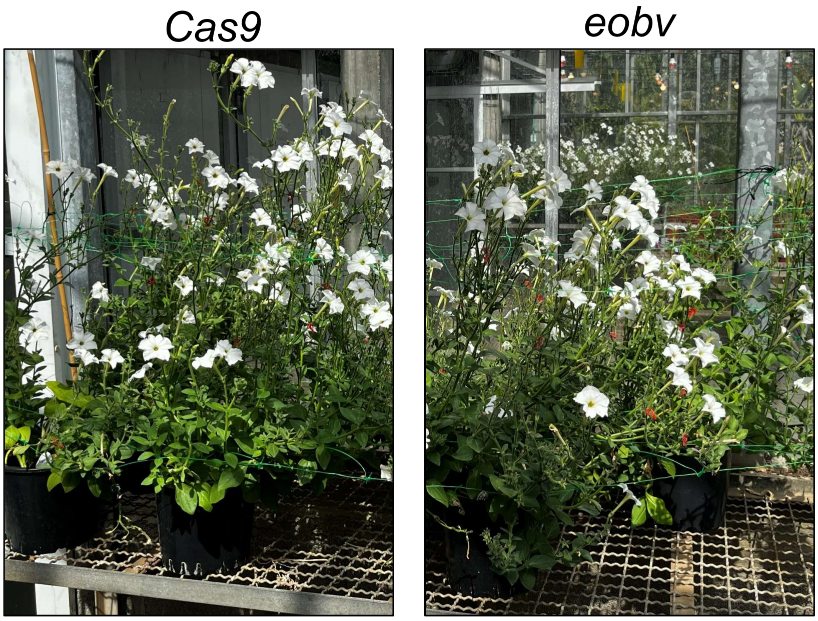

Figure S10. Representative photographs of 2-month-old plants of Cas9 control and *eobv*-knockout lines.

a ***C4H2* (Peaxi162Scf00390g00225):**

ACAATATCCAACTCATAATCTTATCAATCTTGTTTGAGTAATCAACATTAATTCTTATATGC
*ATCTAGTAAGTTCTTTTACAAAAACT**CACCAACC**TTTCCACTATTTCTTTTTCCTCTACCA*
*AACCTCCAATCTACCATCTTATCAACTCCGTCAAACTTAACACCGTTAACCTCCC**CACC***
***TACC**CCCACCAGTTTCCCTCTATATAAATCCACATATACACACCTTTATCAGCCGTACCA*
AACAAACTCACTTTTATTGTCCAAACGAAAAAAAAAAAAAGAACTTGTCCAAACAAAA

***C4H2* (Mitchell):**

ACAATATCCAACTCATAATCTTATCAATCTTGTTTGAGTAATCAACATTAATTCTTATATG
*CATCTAGTAAGTTCTTTTACAAAAACT**CACCAACC**TTTCCACTATTTCTTTTTCCTCTAC*
*CTAACCTCCC**CACCTACC**CCCACCAGTTTCCCTCTATATAAATCCACATATACACACCT*
TTATCAGCCGTACCAAACAAACTCACTTTTATTGTCCAAACGAAAAAAAAAAAAAGAAC
TTGTCCAAACAAAA

***PAAS* (Peaxi162Scf00561g00021):**

AAGTAAGGCTAGTATTCTTTTGTGTAAATCAATCTACCTTACTCATCCACCAAGGTTGA
GATAGTGTGGAATCGGATTTAATAAATATCTATGTCATTTTTTCACATAGAGGTCTTGCA
AATATTTTCAGTACAATTCAATTTTATCATAAGTTACCAAAGGTGTATAGCTGGATCTATA
TATAATTAGATTGCTAAGTTCATGTTGCACATTCATATTATAATACTCTCTTTGTTTGTTC
CCTTCTCTGCTTTTAGTTTAATTGTACGTCATATACCTTCAAATTCAAAATTTATTTCC

***BSMT* (Peaxi162Scf00047g01123):**

TTACTCCAATACCCGTTCGAATTTTGAATCTCCAAAATAAAAGAATTGACTTCAAATGA
GAACACATAAGAATTGAAACAATAACTAAAGCCTCTTACTAAGTTTATAATATAGATTTAG
ACCACTTACTCATCTATATCCCTGTCACTGTCAGGGCCAATACTGGAATTTTAGTGGAC
AAACTTCAGCAGTAGCACCTTTCTATAAATAGGTAATAAATTCTTGCCATGTAGAAGCA
CTATCTCACATAAAGAATAACAAACAAAAAGATCAAGAAAGAGATACTCATAGCAAGAA
GAA

b

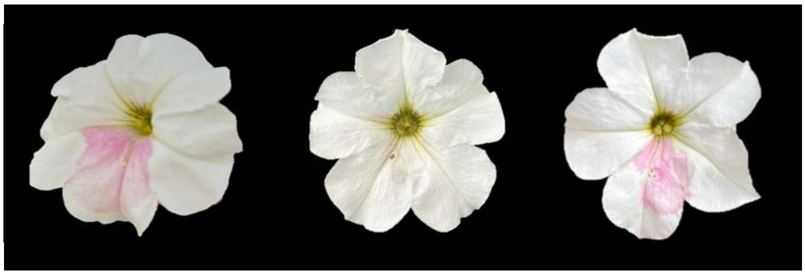

c

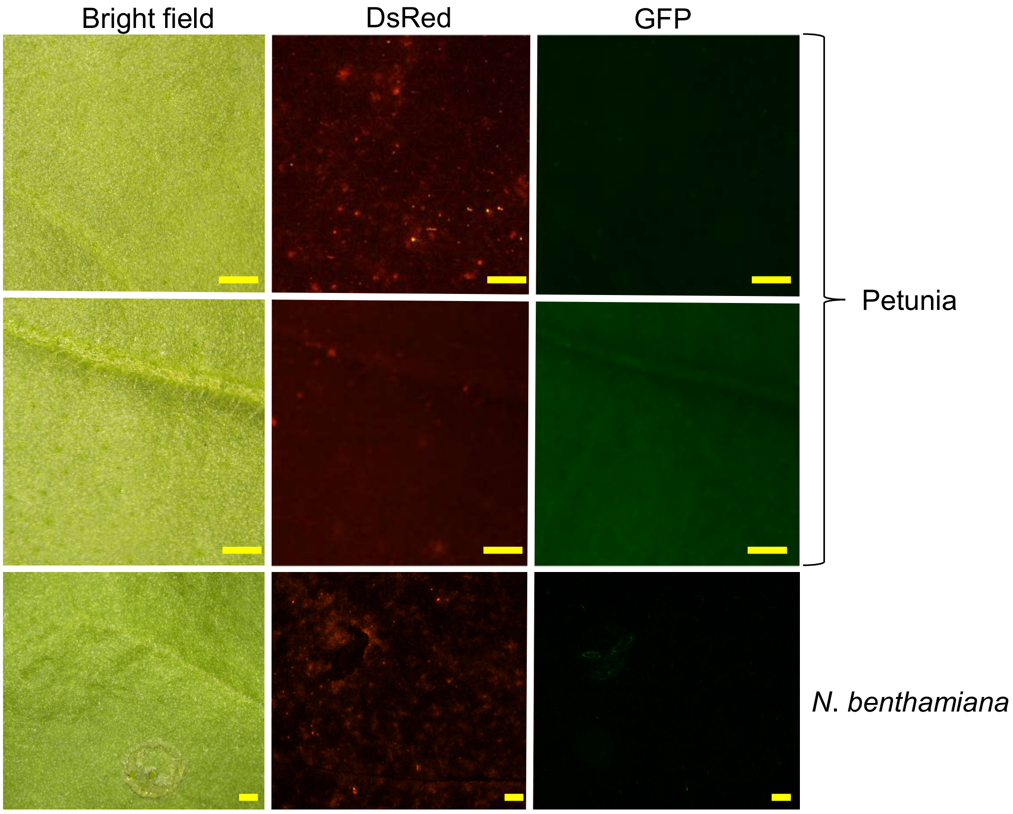

Figure S11. Promoter analysis of scent biosynthesis genes. a) Promoter sequences of *C4H2*, *BSMT* and *PAAS*. Nucleotides marked in red indicate the conserved motif YACCWACY identified by AtMYB4. Sequences were obtained from Sol Genomics Network (https://solgenomics.net/). The sequence of *C4H2* promoter cv. Mitchell was obtained by PCR performed on genomic DNA extracted from flowers followed by Sanger sequencing. **b)** Representative photos of petunia flowers inoculated with agrobacteria carrying C4H2p:*RUBY* (left), C4H2p:*RUBY* together with 35Sp:*EOBV* (middle) and C4H2p:*RUBY* together with 35Sp:*MYBYS* (right). **c)** Representative photos of petunia and *Nicotiana benthamiana* leaves inoculated with agrobacteria carrying C4H2p:*RUBY*, 35Sp:*MYBYS* and AtUbqp(1460):*DsRed.* Bar = 1 mm. C4H2, CINNAMATE 4-HYDROXYLASE 2; PAAS, PHENYLACETALDEHYDE SYNTHASE; BSMT, S-ADENOSYL-L-METHIONINE:BENZOIC ACID/SALICYLIC ACID CARBOXYL METHYLTRANSFERASE.

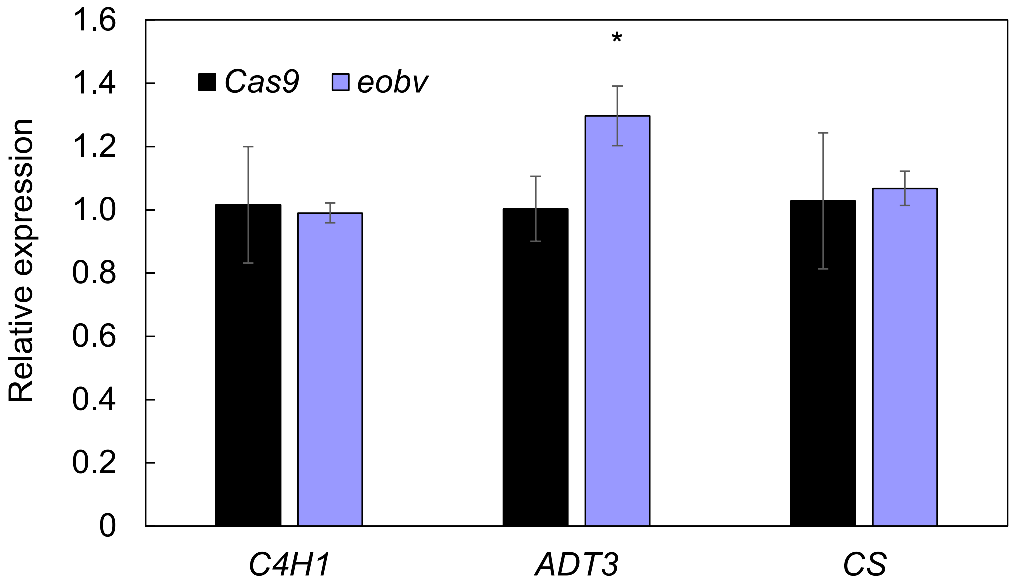

Figure S12. Expression of selected genes containing the YACCWACY motif, as identified in the genome-wide analysis, in *eobv*-knockout lines vs. *Cas9* controls. RT-qPCR analysis was performed on RNA extracted from flowers at 2 days postanthesis. Data are means ± SEM (n = 3). Data were normalized to the geomean of *EF1α* and *UBIQUITIN*. Standard errors are indicated by vertical lines. Significance of differences between treatments was calculated by two-tailed unpaired Student's t-test: *$P \leq 0.05$. C4H1, CINNAMATE 4-HYDROXYLASE 1; ADT, AROGENATE DEHYDRATASE; CS, CHORISMATE SYNTHASE.

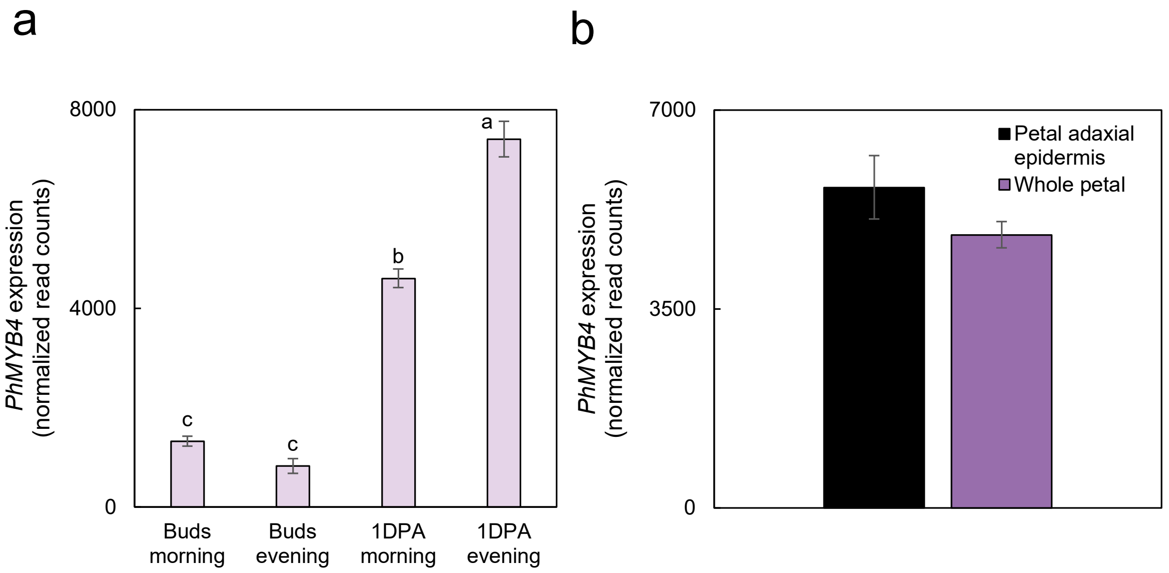

Figure S13. Diel, developmental and spatial expression patterns of *PhMYB4*. a) *PhMYB4* expression levels in petunia floral buds and flowers 1 day postanthesis (1DPA) in the morning (1000 h) and evening (1900 h). Transcripts were detected by RNA-sequencing performed in Shor et al. (2023b). Significance of differences was calculated by Tukey's multiple comparison test following one-way ANOVA. Values with different letters are significantly different at $P \leq 0.05$. **b)** *PhMYB4* transcript levels in the petal adaxial epidermis. Normalized counts in the petal adaxial epidermis from the RNA sequencing performed in Skaliter et al. (2024). Data are means ± SEM ($n = 3$). Standard errors are indicated by vertical lines.

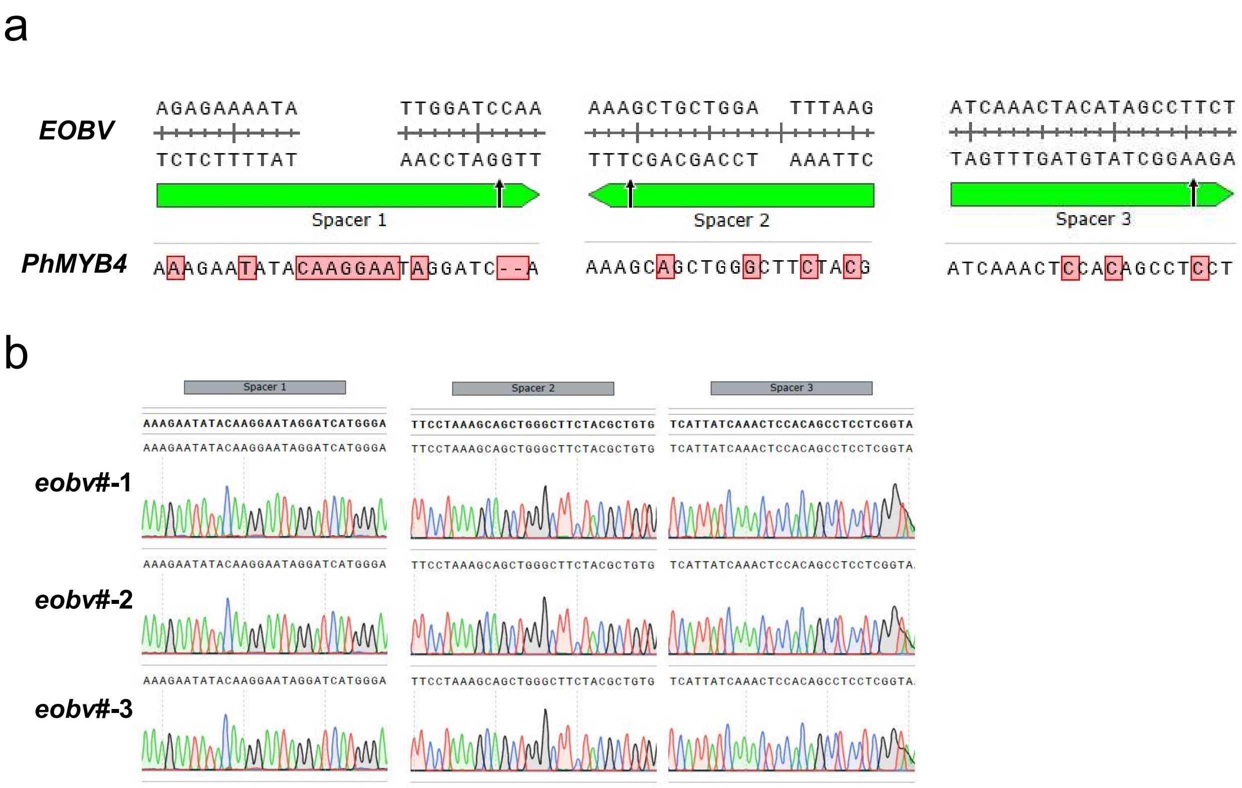

Figure S14. ***PhMYB4* genomic sequence is not affected by knockout of *eobv*. a)** Alignment of spacer sequences designed to target *EOBV* and *PhMYB4* genomic sequence.
b) Sanger sequencing of *PhMYB4* in *eobv*-knockout lines #1–3 showing no editing events. Gray rectangles mark the location of the *PhMYB4* sequences with the highest homology to the spacers designed to target *EOBV*.

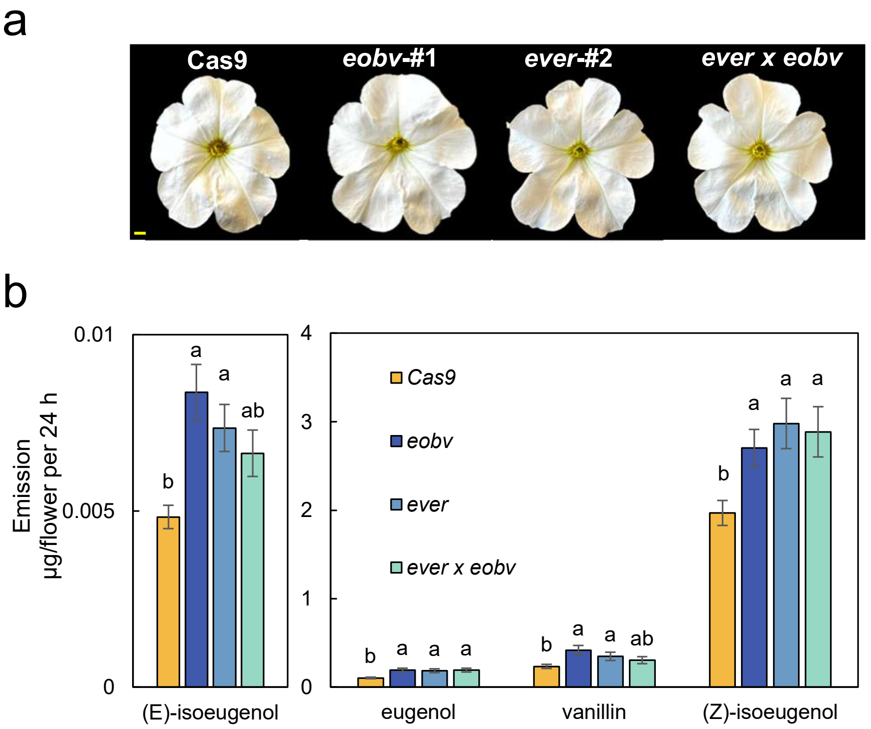

Figure S15. Stacking of *ever* and *eobv* knockout alleles. a) Representative petunia cv. Mitchell flowers from *Cas9* control, *eobv*-#1, ever-#2 and *ever* x *eobv* at 2 days postanthesis (2DPA). Bar = 1 cm. **b)** Dynamic headspace (collected for 24 h and analyzed by GC–MS) was performed on flowers at 2DPA collected from *Cas9* (control), *ever-#2*, *eobv-#1* and *ever* x *eobv.* Data are means ± SEM (*n* = 15–17). Significance of differences was calculated by Tukey's multiple comparison test following one-way analysis of variance. Values with different letters are significantly different at $P \leq 0.05$. Standard errors are indicated by vertical lines.

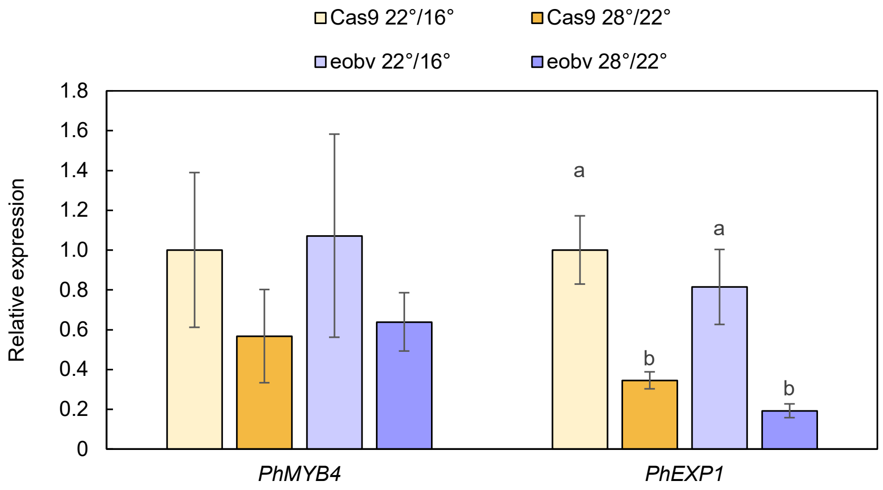

Figure S16. Effect of elevated growth temperature regime on expression of *PhMYB4* and α-*expansin* (*PhEXP1*) in *eobv*-knockout lines. Plants of *eobv*-knockout lines and control *Cas9* were grown for 1 month in a phytotron at different growth temperatures: 22/16°C and 28/22°C. RT-qPCR analysis was performed on RNA extracted from flowers at 2 days postanthesis. Data are means ± SEM (n = 4–5). Data were normalized to the geomean of *EF1α* and *UBIQUITIN*. Standard errors are indicated by vertical lines. Significance of differences was calculated by Tukey's multiple comparison test following one-way ANOVA. Values with different letters are significantly different at $P \leq 0.05$.

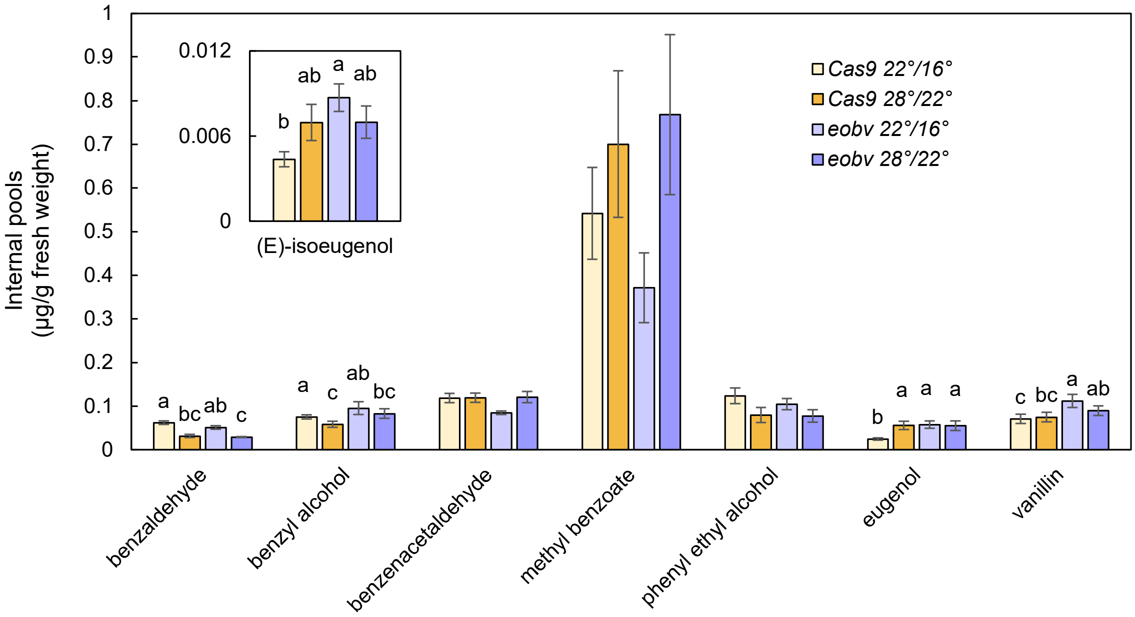

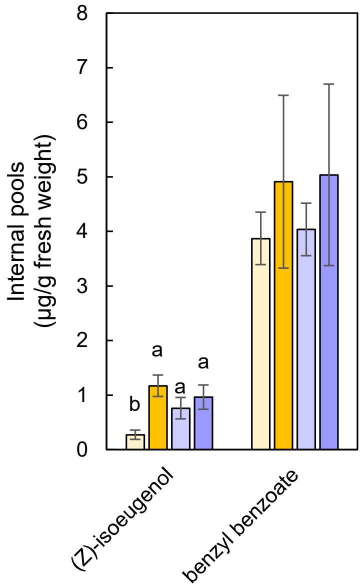

Figure S17. Effect of elevated growth temperature regime on internal pool levels of *eobv*-knockout lines compared to control Cas9. Plants were grown for 1 month in a phytotron at 22/16°C or 28/22°C. Internal pools were extracted from flowers 2 days postanthesis of *Cas9* (control) and *eobv* lines at 2000 h followed by GC–MS analysis. Data are means ± SEM (n = 9–15). Significance of differences was calculated by Tukey's multiple comparison following one-way ANOVA or Steel-Dwass All-Pairs test. Values with different letters are significantly different at $P \leq 0.05$. Standard errors are indicated by vertical lines.
